# Introductory Lecture to the Tenth Annual Course of Instruction in the Chicago Medical College, October 5th, 1868

**Published:** 1868-11

**Authors:** N. S. Davis

**Affiliations:** Professor of Principles and Practice of Medicine in Chicago Medical College, and of Clinical Medicine in Mercy Hospital


### CHICAGO MEDICAL EXAMINER.

N. S. DAVIS, M.D., Editor.

VOL. IX. NOVEMBER, 1868. NO. 11.

### Original Contributions.

### ARTICLE XLIV.

### INTRODUCTORY LECTURE TO THE TENTH ANNUAL COURSE OF INSTRUCTION IN THE CHICAGO MEDICAL COLLEGE,

October 5th, 1868.

By N. S. DAVIS, M.D., Professor of Principles and Practice of Medicine in Chicago Medical College, and of Clinical Medicine in Mercy Hospital.

Gentlemen:—It has become my duty, in behalf of the Faculty, to extend to you the customary greeting at the commencement of this, the Tenth Annual Course of Instruction in this Institution.

The occasion, though of annual recurrence, is nevertheless one of marked interest to each one of you. Each period of human life has its pains and its pleasures; its toils, its responsibilities, and its rewards. But none of these periods is more pregnant with importance than that which is more especially devoted to a preparation for the active duties of Life, usually called the period of pupilage. It is during this period that the young man not only acquires the knowledge which is to enable him to take his place among the active workers in society, but he also forms and fixes those habits, and develops those mental activities, that determine with much certainty both the position he shall attain in his calling, and the degree of his usefulness in society. Hence, whatever relates to your habits, your mental discipline, and your acquisition of knowledge, now, is of vital importance in its bearing upon all the future of your lives.

If you spend this time in the mere passive reception of such facts and items of knowledge as may be presented to you, without critically examining each item and measuring its relations to all others, you may close your nominal period of pupilage with a show of knowledge, but without the mental discipline to apply it accurately, or the mental activity that continually demands an increase of acquisition. Your mental growth will, consequently, stop at the threshold of your professional career; and though such career should be extended over thirty or forty years, yet at the end you would be found still working on the same scanty collection of materials that supplied you the first year, and following out the same monotonous routine of practice in which you started.

These simple truths cannot be too strongly impressed on the mind of every young man entering upon the study of medicine with the intention of engaging in the active duties of practice. The extent of the field of medical sciences to be mastered; the number and variety of the conditions influencing human life and health, and the intricate nature of the questions involved in the diagnosis and treatment of disease, all demand of the medical student who would do justice, either to his calling or his conscience, a capacious, well-stored mind, disciplined to the highest degree of activity and exactness.

Yet it requires but a casual acquaintance with the great body of practitioners in our country, to perceive that their most prominent deficiencies, and most frequent embarrassments, arise directly from the defective development of the mental qualities just named. And this, again, is easily traceable to the peculiarities of the system of medical education which has prevailed in this country since our existence as an independent nation. Theoretically, that system has its basis in a course of three years of quiet study in the office of a private preceptor, aided by a short annual review of the several branches in the medical colleges. And if it was true, that every practitioner who receives students into his office, faithfully acted the part of a preceptor or teacher, by judiciously directing which branches should be studied first, and by daily systematic examinations and instructions of the student in those branches; and if the organization of the colleges was such that each student was required to confine his attention to the reviews and demonstrations in the branches corresponding with his period of advancement in study, there would be little fault to find with the system. For while it would make good students, on one hand, on the other it would contribute largely to keep up the habits of reading and close observation by the practitioner acting as preceptor.

Practically, however, it has long since become the custom for students to enroll their names in the office of some practitioner, and spend the period of private pupilage in the desultory reading of medical books, without an hour of instruction from their so-called preceptors once a month; while the colleges are so organized that no classification of the pupils is allowed, the new beginner and the advanced student being mixed in the same lecture-room, and subjected alike to the hearing of a didactic review of all the branches of medicine, heterogeneously, within the period of sixteen or eighteen weeks. The young man, who thirty or sixty days since left the plough, the workshop, or the schoolroom, and who does not yet know enough of the elements of medical science to tell the number of bones in his own cranium, and the student of two and three years, are placed side by side, and made to listen daily, at alternate hours, to lectures on the elements of anatomy and practical medicine, physiology and the details of practical surgery, chemistry, and obstetrics, etc., at such a rate that in the short period of four or five months they have had poured into their ears instruction in anatomy, physiology, chemistry (organic and inorganic), materia medica, histology, microscopy, general pathology, pathological anatomy, hygiene, medical jurisprudence, practical medicine, surgery, obstetrics, and clinical observations at the bedside.

When we remember that the careful reading of a creditable text-book on any three of these branches of medical science would occupy a good student more than the eighteen weeks allotted by most of the colleges for an entire lecture term, we shall realize how absurd the present system appears in actual practice. Indeed, it is doubtful whether human ingenuity could devise, by the most careful deliberation, a scheme of professional education better calculated to prevent the acquisition of extensive knowledge, thoroughness of mental discipline, or quickness and accuracy of observation; or one more directly favorable to superficial and imperfect acquirements, hasty and illogical reasoning, and the abrogation of all distinction between the elementary and the practical—the foundation and the superstructure.

So glaring were the defects in our system of medical education, and so prominent the evils resulting, both to the profession and the community, that more than thirty years since, it began to be most severely criticised by such men as Nathaniel Chapman, Daniel Drake, Samuel Jackson, Alexander H. Stevens, and many others of high standing in the profession. From 1835 to 1846, the subject not only engaged the attention of many of the most eminent men connected with the medical colleges, but elicited earnest discussion in most of the State Medical Societies then existing. These discussions resulted in the assembling of the first National Medical Convention, held in the city of New York, in May, 1846; and from which originated the American Medical Association. Since the organization of that Association, not an annual session has passed during which there was not a general admission on the part of its members from every part of the country, that our system of medical college instruction is most lamentably defective. And even in the Convention of delegates from medical colleges alone, held in Cincinnati, May, 1867, not a single member was found willing to attempt a defence of the present system.

Yet such has been the force of self-interest, and such the overpowering influence of the desire on the part of each college to swell the size of its classes and the number of its alumni, coupled with a want of confidence in the faithful coöperation of competing colleges, that no essential changes in the system have been effected. It is true that new branches have been added to the college curriculum; a few additional professorships have been created; from two to four weeks have been added to the annual lecture terms in most of the colleges; and additional clinical facilities have been provided in many places; but the same ignoring of preliminary education; the same neglect of all classification of students or successive order of teaching; and the same repetitional, heterogeneous, intermingling of instruction on all branches of medical science and art, to all grades of students in one class, in the short space of four or five months, still exists in all but one or two of the medical colleges in our country.

Within the last few years the faculties of many of the colleges, in different parts of the country, have officially signified their readiness to adopt such specified changes as would remedy all the important defects of the present system, provided such changes can be adopted simultaneously by all, or a large majority, of the colleges in all parts of the country. But the faculties of many others remain silent, while the organs representing a few, let no opportunity pass unimproved, to ridicule whoever earnestly advocates improvements demanded alike by the interests of humanity and the honor of our noble calling. They are careful never to attempt a defence of the present defective system of education, or manfully to criticise proposed plans for improvement. But they are ever ready to sneeringly apply the epithet "*Pseudo-Reformers*," and to talk facetiously of "*Apostles of Reform*," and "*Phantoms in Black*." This small class of small men always remind us of one of the finny tribes, which, incapable of any other defence, when attacked expels from its ink-bag a mass of black fluid by which the water is rendered so turbid that its own body is hidden from the view of its assailant.

I need not remind you, gentlemen, that the members of the Faculty of this College are among the most earnest advocates of such changes in the general system of medical college instruction, as are almost universally admitted to be necessary, for they have demonstrated this in the organization of the College itself.

So early as 1857, while three of the present Faculty of this College held prominent chairs in the Faculty of the Rush Medical College of this city, chiefly through the active advocacy of Professor Byford and myself, a plan was devised for extending the annual college term of that institution to six or eight months, classifying the students, and arranging the order of teaching into junior and senior departments, which received the sanction of a large majority of the, then existing, faculty, and was finally prevented from practical adoption solely by the opposition of the late Professor Brainard, who was President of the College. Two years later, namely, in the spring of 1859, a very favorable opportunity occurred for the establishment of a new medical college in this city, under the auspices of the Board of Trustees of what was then called "Lind University." Professor H. A. Johnson, who had previously resigned his position in the Rush Medical College, and Professor E. Andrews, acting more or less in concert with Professor R. N. Isham and the late Dr. David Rutter, ascertaining that the said Board of Trustees were desirous of establishing a Medical Department of their University, and were willing to sanction such an organization as the interests of the profession and the community required, without regard to effete customs or the mere number of students, entered into a contract with the Board, by which they were authorized to nominate a Faculty, and adopt such plan of organization as the faculty so nominated might deem proper.

Having thus taken the initiatory steps for the organization of a new medical school, Professor Johnson, in behalf of the parties just named, communicated the fact to my colleague in the chair of Obstetrics, and to myself, together with an invitation for us to join them, and aid in the completion of the organization. Having fully satisfied ourselves that no important changes could be effected in the plan of instruction in the Rush Medical College, and regarding such changes as of far more importance to the profession than any mere personal interests of our own, we cheerfully resigned our places and entered upon the new work. A plan of organization for the new school was speedily matured, embracing thirteen professorships; the principle of progressive study and teaching, by dividing the various branches into junior and senior groups or departments, and classifying the students accordingly; the requirement of annual examinations; the adoption of hospital clinical instruction as an essential part of the course; and the extension of the lecture term.

To demonstrate to all the profession, that those engaging in the organization of the new college were not actuated by any petty personal rivalry, or desire to establish a new school merely to compete with those previously existing for numbers of students, regardless of the great principles of education, the lecture term adopted was five calendar months, and the annual lecture fees fifty dollars cash and no credit, while at that time the Rush Medical College, in our own city, had a lecture term of only *sixteen weeks*, and an annual lecture fee of only thirty-five dollars, and our next nearest neighbor, the Medical Department of the University of Michigan, charged only an initiatory fee of ten dollars.

Having completed the plan of organization and nominated competent men to fill the several chairs, the same received the sanction of the Trustees of the University. Rooms for temporary occupancy were fitted up in what was called Lind's Block, on Market Street, and on the first Monday in October, 1859, the first course of lectures in this institution was commenced, with a class of about thirty students. With the Mercy Hospital as an ample field for daily clinical instruction, the entire course was completed with regularity and great satisfaction. At the first public commencement, on the first Monday in March, 1860, the degree of M.D. was conferred on nine regular students.

Three years subsequently, the ground was purchased, and the building in which we are now assembled was erected, and dedicated as a temple for the advancement of medical science and art.

About the same time, Mr. Sylvester Lind, after whom the University had been named, having failed, from pecuniary reverses, to endow the Theological Department, as he had originally agreed to do, the Board of Trustees procured a change of name to that of Lake Forest University. This change in the name, together with the failure of the Board to furnish the money for the new college building in accordance with the original agreement, led to the organization of a new Board of Trustees under the laws of the State in which the title to the Medical College property was vested, and the new corporation took the name of the Chicago Medical College.

From the first year of our organization to the present time, the prosperity of the College has been remarkably uniform and satisfactory. Commencing with thirty-three students and twelve graduates (including three *ad eundems*) the first year, we closed our last and ninth course with 113 students and fifty graduates (including two *ad eundem* and three honorary degrees).

In pecuniary matters our condition is equally satisfactory. Instead of a load of indebtedness taxing the pockets of every member of the Faculty annually to pay the interest; or a joint-stock concern, in which every chair is encumbered with one or more shares of the stock, the Trustees are the absolute owners of the College building and grounds, and completely secured from all encumbrances. In addition, we have accumulated an admirable museum for illustration of the various branches; a library of several hundred volumes; and a complete laboratory for instruction in elementary, organic, and practical chemistry.

Having, in the short period of nine years, secured all these results, and fully demonstrated the success and permanency of our College, the Trustees and Faculty have unanimously agreed to extend our annual lecture term to six months; to complete the classification of studies and students, by making three departments corresponding with the three years of study; to require three annual courses of lectures before graduating, with a thorough examination at the close of each; and to exact at least a moderate standard of preliminary education at the entrance.

At the same time, they have increased the facilities for clinical instruction, added a separate chair on Public Hygiene, provided special courses on Ophthalmology, Orthopædic appliances, physical and instrumental diagnosis, and Comparative Anatomy; with the most perfect arrangements for detailed instruction in practical and analytical chemistry. And at the opening of this, the tenth annual course of instruction, it is with unfeigned pleasure and gratitude that I welcome you, gentlemen, to the halls of the first American Medical College whose organization is in accordance with those principles of edcation everywhere acknowledged to be correct; whose system of instruction, both in regard to length of term and systematic order of studies, is commensurate with the field of medical science and the demands of the profession; and whose material appliances are complete in every part. I acknowledge a feeling of pride that I am identified with an institution whose Trustees and Faculty have risen so far above the mere considerations of pecuniary gain and petty competition for numbers of students, as to demonstrate to the whole profession of our country the practicability of establishing and maintaining medical colleges on a basis commensurate with the wants, the interests, and the honor of the profession.

And I am sure there is not one of you, who coöperate with us by submitting as students to a patient, systematic, and thorough course of professional study, who will not, in after years, feel an equal, nay! a greater pride in your Alma Mater.

Her successful example must and will be followed, sooner or later, by other colleges in every part of the country. Many of those now existing may cling to their idols, namely, jealousy of each other and an overweening desire for large classes, regardless of quality; but if so, they will some day wake up to find themselves supplanted by new ones on a more rational basis. The change may be slow. Indeed, all great improvements in human institutions are of slow growth.

The revolution may destroy the work of centuries in a day; but, like the whirlwind, it leaves only wrecks and debris in its path, which often require generations to remove and replace with improved organizations.

But, fellow-students, having indulged in this brief glance at the past history of the Chicago Medical College, and welcomed you to its halls, I must not close without a few words to you regarding the duties and responsibilities devolving upon every student of medicine. If there are any established truths in the various departments of medical science; if there is any real efficacy in the action of medicines on the human system; if their intelligent administration is capable of mitigating human suffering and prolonging human life; if the surgical art, skilfully applied, is capable of rectifying deformities, removing obstructions and tumors, staying the flow of blood, and healing wounds; in few words, if the medical profession is a necessary part of every civilized society, its members the conservators of public health and the welcome guests at every bedside of the sick, then is there a responsibility resting upon every student which he cannot appreciate too early or too clearly.

You are preparing to enter upon the practice of a profession where every step you take is in relation to the two most sacred interests of your fellow-men—namely, life and health. On the extent and accuracy of your knowledge, the quickness of your perceptions, the coolness of your judgment, and the skill of your manipulations, may depend the life of the first mother or child with whom you have to do. Medical science is constituted of such facts, principles, and materials from all other sciences, as relate to the human system, in all its varying conditions of health and disease. Medical art the application of this vast aggregation of facts, principles, and appliances, to the prevention and treatment of all the diseases, accidents, and imperfections to which the human family are liable.

The field open for cultivation by the medical student is exceedingly broad; the three years' time allotted for improvement is comparatively short; and he who expects to reap a harvest of either usefulness or honor, will find no time to loiter by the way. He cannot afford to lull his senses with the fumes of tobacco, or poison his blood and pervert his passions with alcohol. He cannot recline on the alluring sofa of sensual pleasures, nor waste time in the lecture-room listening to the facetious anecdotes or vulgar stories of his pretended teachers. His work is an earnest and responsible one. And all the more so, because a large majority of those with whom he has to do, are incapable of judging whether he does them justice or not. At every step, he requires accuracy of knowledge, quickness of perception, purity of thought, correctness of deduction, and promptness of action.

My hearers, these are qualities that are neither born with us, nor furnished ready made by any institution; nor can they be purchased with any quantity of money or good wishes. They are, however, within the reach of all of you. Patient, persevering, systematic study, animated by the noble aspiration to be useful in your day and generation, and associated with a daily life of unbending virtue, will develop every one of them in their fulness. It should be the aim of every student of medicine to so qualify himself, both in the acquisition of knowledge and in the development and discipline of his mental faculties, that he can give to every patient committed to his charge, all the benefit that the science and art of medicine is capable of affording.

Gentlemen, if you will treasure up, and faithfully act, through life, on these brief and hastily-expressed hints, you may not all gather an abundance of that wealth which is measured either by gold or *greenbacks*, but you will surely gather what is far better, a consciousness of having made the world better and happier for your having lived in it, and a conscience void of offence towards God and man.

---

CHLORIDE OF AMMONIUM AND TINCTURE OF ACONITE IN OVARIAN NEURALGIA.—Dr. J. Waring-Curran states that this combination seems to have a magical influence in the treatment of ovarian neuralgia. He reports six cases in which various sedatives and anodynes had been tried in vain. He prescribed an eight-ounce mixture, containing two drachms of muriate of ammonia, with five-drop doses of tincture of aconite, and found that before the mixture was finished by the patient the pains had entirely ceased.—*Medical Press and Circular*, August 19, 1868.

